# *DHXT1*, a Virulence Factor of *Dactylellina haptotyla*, Regulates Pathogenicity by Participating in Trap Formation and Metabolite Synthesis

**DOI:** 10.3390/ijms25137384

**Published:** 2024-07-05

**Authors:** Xing-Fu Wen, Ting-Ting Shi, Ya-Qi Zhang, Si-Han Wang, Chun-Mei Xiang, Pei-Ji Zhao

**Affiliations:** State Key Laboratory for Conservation and Utilization of Bio-Resources in Yunnan, School of Life Sciences, Yunnan University, Kunming 650091, China; wenxingfu0925@163.com (X.-F.W.); ttoymyy@163.com (T.-T.S.); 18213958670@163.com (Y.-Q.Z.); sihanwang0028@163.com (S.-H.W.); chunmeix2022@163.com (C.-M.X.)

**Keywords:** virulence factor, *Dactylellina haptotyla*, *CAP10*, adhesive knob formation, metabolites

## Abstract

The capsule-associated protein 10 gene (*CAP10*) is indispensable due to its involvement in pod formation and virulence maintenance in *Cryptococcus neoformans*. The function of the *CAP10* gene in nematode-predatory fungi remains unreported. As a typical nematode-trapping fungus, *Dactylellina haptotyla* efficiently captures nematodes using adhesive knobs, which has potential applications in the biological control of plant-parasitic nematodes. In this study, we investigated the function of DHXT1 (a CAP10 homologous protein) in *D. haptotyla*–nematode interactions based on the disruption and overexpression of *DHXT1*, phenotypic analysis and metabolomic analysis. As a result, it was shown that the disruption of the *DHXT1* gene causes a marked decrease in the number of adhesive knobs, and on the contrary, the overexpression of the *DHXT1* gene causes a substantial increase in the number of adhesive knobs. Interestingly, the variety of metabolites increased with the disruption of the *DHXT1* and decreased with the overexpression of the *DHXT1* gene. The results suggest that *DHXT1* effects pathogenicity through its involvement in adhesive knobs’ formation and metabolite synthesis and serves as a key virulence factor in *D. haptotyla*.

## 1. Introduction

Plant-parasitic nematodes cause dramatic yield and economic losses to crops worldwide annually, either through direct damage to the host or by being virus vectors [1]. Nematicides are the most toxic insecticides used in agriculture [2], and their high broad-spectrum toxicity not only severely damages the environment and agro-ecosystems, but also has the side effect of reducing soil fertility and disintegrating the soil [3]. Furthermore, the frequent use of chemical nematicides leads to the development of nematode resistance, which is even more detrimental to nematode management [4]. Biological control is not only an environmentally friendly way to manage plant diseases, but also improves soil fertility without affecting animals and plants and is regarded as an ideal alternative to chemical nematicides [5]. Natural enemy organisms of plant-parasitic nematodes include fungi, bacteria, viruses, rickettsiae, actinomycetes, vortex worms, etc., among which, nematode-trapping fungi (NTF) are one of the major regulators of nematode density and act as a very vital natural control of plant-parasitic nematode populations in nature. As natural enemies of nematodes [6], NTF sense host signals and specialize mycelia into traps such as constricting rings (CRs), adhesive columns (ACs), adhesive networks (ANs) and adhesive knobs (AKs) to trap and kill nematodes [5]. Nematode predation by NTF is a multifactorial and coordinated process that involves attraction and recognition, adhesion, penetration and digestion [7]. Given the potential application of NTF as a biocontrol agent, in recent years, the exploitation of nematicidal biologics with NTF has been gradually emphasized and has been the subject of an increasing number of studies. NTF utilize a variety of methods to capture and digest nematodes. Most NTF wait passively for nematode contact after soil colonization; thus, some NTF produce compounds that attract nematodes to increase trapping opportunities [8]. For example, *Arthrobotrys oligospora* produces methyl 3-methyl-2-butenoate to attract nematodes [9], while *Duddingtonia flagrans* attracts nematodes by producing 6-methyl salicylic acid [10]. In addition, *A. oligospora* produces a variety of specialized enzymes to immobilize and digest nematodes [11,12,13]. 6-Methylsalicylic acid, m-cresol and C-280 produced by *A. oligospora* were found to have nematicidal activity [14,15].

As a typical NTF, *D. haptotyla* captures nematodes and enters the parasitic stage by producing adhesive knobs. Comparative genomic analyses suggest an evolutionary trend toward morphological simplicity and increased efficiency in adhesive knobs’ formation [16,17]. Moreover, studies have shown that of the four traps, CR, AN, AC, and AK, AK is the most effective at capturing nematodes and that a single sticky ball is usually sufficient to capture large nematodes [16]. Although mucus balls have a simple morphology, the adhesion layer of up to 20 μm on their surface is the key to their efficient capture of nematodes [16]. It was shown that 23 genes encoding adhesion proteins were significantly upregulated during nematode capture by AK [16]. In addition, the disruption of genes encoding adhesion proteins in *A. oligospora* significantly reduced the ability of NTF to capture nematodes [18]. Additionally, it has been found that *D. haptotyla* has more stable population densities and trap incidence in different environmental soils and suppresses plant root-knot nematode diseases to a certain extent [19,20]. A large number of secondary metabolites were significantly up-regulated during the capture of nematodes by *D. haptotyla* YMF1.03409, among which, 2-furoic acid had strong nematicidal activity [21,22].

The pathogenic fungus *Cryptococcus neoformans* causes systemic mycosis in animals and humans and affects the central nervous system of AIDS patients, causing meningoencephalitis [23,24]. The formation of polysaccharide capsules, the main known virulence factor produced by *C. neoformans*, is closely linked to four capsule-associated genes (*CAP10*, *CAP59*, *CAP60*, *CAP64*) [25]. It was shown that the knockout of *CAP10*, which has homology to xylosyltransferase [26], leads to the failure of polysaccharide capsule formation, and this did not show pathogenicity in animal models. However, the virulence of this strain was restored with the complementation of the *CAP10* gene [24]. There are few studies on CAP-related genes in NTF; therefore, the following study will investigate the role of *CAP10*-related genes in the *D. haptotyla*–nematode interaction process. Xylosyltransferase encoded by *CXT1* in *C. neoformans* [26] and an enzyme named rumi in *Drosophila* [27] were retrieved using blastn with the genome of *D*. *haptotyla*, and six CAP10-related proteins were obtained, and one of the genes was selected and named *DHXT1*. This study was designed to investigate the effect of *DHXT1* on mycelial growth, conidial germination, trap formation, pathogenicity and metabolites of *D*. *haptotyla* YMF1.03409.

## 2. Results

### 2.1. Sequence and Phylogenetic Analysis of DHXT1

A total of six homologous proteins were screened from the genome of *D*. *haptotyla* based on CXT1 in *C. neoformans* and rumi in *Drosophila* and named EVM03 G008080.1, EVM00 G010740.1, EVM01 G010500.1, EVM09 G002510.1, EVM00 G010740.1 and EVM00 G006560.1 as DHXT1-6. DHXT1, containing CAP10 domain and glycosyltransferase 90 family structural domains, was selected to study its biological function in *D. haptotyla* YMF1.03409. The biological function of DHXT1 containing CAP10 domain and glycosyltransferase 90 family structural domains in *D*. *haptotyla* was further investigated. The results (Figure S1) showed that CAP10 homolog proteins from predatory nematode fungi are a relatively distinct branch in the phylogenetic tree, implying that these CAP10 homolog proteins from NTF are distinct from CAP10 from other fungi.

### 2.2. DHXT1 Is Involved in Mycelial Growth and Does Not Affect Conidial Germination and Mycelial and Trap Morphology

**Figure 1:**
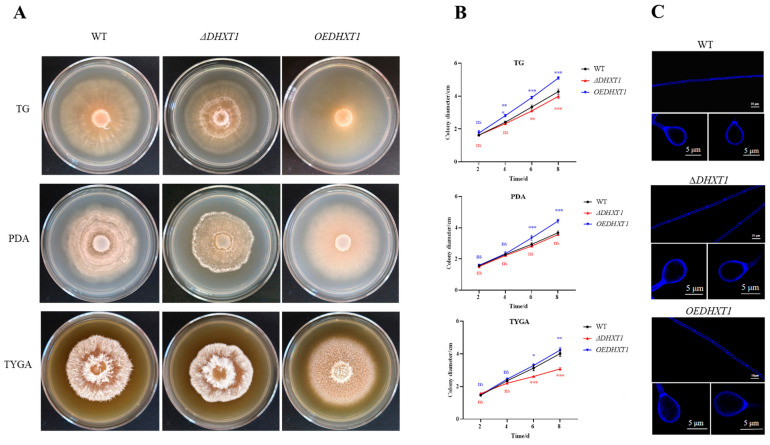
Effect of DHXT1 on mycelial growth. (**A**) Growth of WT, *ΔDHXT1* and *OEDHXT1* transformants on PDA, TG and TYGA plates for 8 days. (**B**) Growth curves of WT, *ΔDHXT1* and *OEDHXT1* transformants on PDA, TG and TYGA plates for 8 days (ns *p* > 0.05, * *p* ≤ 0.05, ** *p* ≤ 0.01 and *** *p* ≤ 0.001). The growth rate of *ΔDHXT1* mutants on PDA, TG and TYGA media was slightly slower than that of the WT, whereas the growth rate of the *OEDHXT1* transformants was faster than that of the WT. (**C**) Mycelial and knob morphology of WT, *ΔDHXT1* mutants and *OEDHXT1* transformants on PDA plates for 6 days after calcofluor white (CFW) staining.

The *ΔDHXT1* mutants and overexpression *DHXT1* (*OEDHXT1*) transformants were obtained by PCR amplification verification (Figure S2). The results revealed that the growth rate of *ΔDHXT1* mutants on PDA, TG and TYGA media was slightly slower than that of the WT (Figure 1A,B), whereas the growth rate of the *OEDHXT1* transformants was faster than that of the WT in all cases (Figure 1A,B). However, fluorescence microscopy results indicated that neither the knockout nor overexpression of *DHXT1* altered the mycelial and knob morphology (Figure 1C). Interestingly, the results showed that the overexpression of *DHXT1* resulted in a significant reduction in aerial mycelia (Figure 1A), whereas the knockout of *DHXT1* did not affect aerial mycelium production (Figure 1A). Furthermore, neither the knockdown nor overexpression of *DHXT1* had any effect on the conidial germination rate, which was 96.23%, 93.06% and 96.68% (Figure S3) for WT, *ΔDHXT1* mutants and *OEDHXT1* transformants, respectively.

### 2.3. Alterations in DHXT1 Do Not Change the Resistance of D. haptotyla

**Figure 2:**
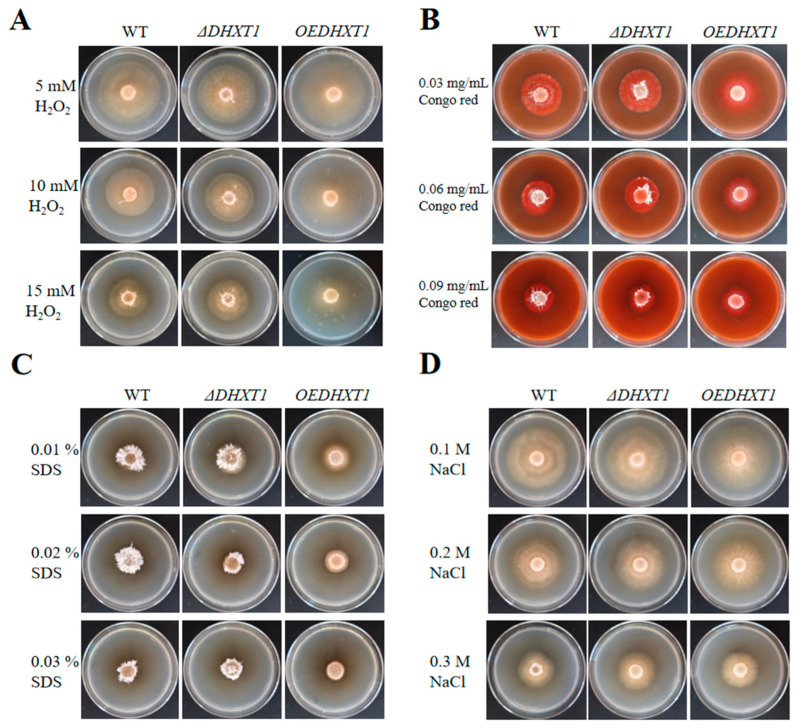
WT, *ΔDHXT1* mutants and *OEDHXT1* transformants growth on TG plates containing different stress conditions. (**A**) WT, *ΔDHXT1* mutants and *OEDHXT1* transformants were cultured on TG plates containing 5, 10 and 15 mM of H_2_O_2_. (**B**) WT, *ΔDHXT1* mutants and *OEDHXT1* transformants were cultured on TG plates containing 0.03, 0.06 and 0.09 mg/mL of Congo red. (**C**) WT, *ΔDHXT1* mutants and *OEDHXT1* transformants were cultured on TG medium containing 0.01, 0.02 and 0.03% of SDS. (**D**) WT, *ΔDHXT1* mutants and *OEDHXT1* transformants were cultured on TG plates containing 0.1, 0.2 and 0.3 M of NaCl.

The WT, *ΔDHXT1* mutants and *OEDHXT1* transformants were maintained on TG plates containing a range of different stress conditions to evaluate their resistance. The results showed that neither the knockout nor overexpression of *DHXT1* significantly altered tolerance to osmotic agents (NaCl), oxidizing agents (H_2_O_2_), and cell wall disruptors (Congo red and SDS). The results showed that there was no statistical difference in the growth of *ΔDHXT1* mutants on TG plates containing different concentrations of NaCl, H_2_O_2_, Congo red, and SDS compared with the wild type (Figure 2A–D and Figure S4), except for 0.3 M of NaCl, on which the *ΔDHXT1* mutants obviously grew faster than the WT (*p* ≤ 0.05). While the growth rate of the *OEDHXT1* transformants was faster than that of the WT in different concentrations of 0.01% of SDS, 0.1 M of NaCl, 0.03 mg/mL of Congo red and 5, 10 and 15 mM of H_2_O_2_, it was not significantly different from the wild type under other conditions (Figure 2A–D and Figure S4). In addition, the results demonstrated that overexpression of the gene led to a reduction in aerial mycelia (Figure 2A–D).

### 2.4. DHXT1 Involved in Trap Formation and Pathogenicity

**Figure 3:**
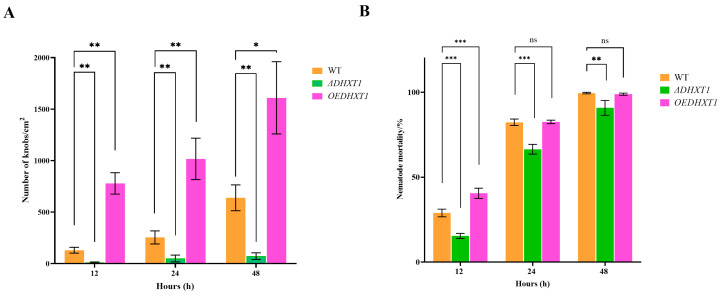
Effect of *DHXT1* on trap formation and nematode mortality. (**A**) The number of knobs in WT, *ΔDHXT1* mutants and O*EDHXT1* transformants after 12, 24 and 48 h of nematode addition. (**B**) Nematode mortality of WT, *ΔDHXT1* mutants and *OEDHXT1* transformants after 12, 24 and 48 h of nematode addition. (ns *p* > 0.05, * *p* ≤ 0.05, ** *p* ≤ 0.01, and *** *p* ≤ 0.001).

The results revealed that the *DHXT1* was involved in adhesive knobs’ formation. The average number of adhesive knobs produced by WT was 130, 255 and 639 knobs/cm^2^ (Figure 3A) after the addition of nematodes induced for 12, 24 and 48 h. The knockout of *DHXT1* led to a significant decrease in the number of traps to 50 and 73 knobs/cm^2^ (Figure 3A) after the addition of nematodes for 24 and 48 h, whereas the overexpression of *DHXT1* caused an increase in traps to 839, 995 and 1761 knobs/cm^2^ (Figure 3A) after the addition of nematodes at 12, 24 and 48 h. The nematode mortality results of WT, *ΔDHXT1* mutants and *OEDHXT1* transformants showed that after 12 h of nematode addition, the nematode mortality of the *ΔDHXT1* mutants (15.37%) (Figure 3B) was decreased compared to that of the WT (28.89%) (Figure 3B), whereas the nematode mortality of the *OEDHXT1* transformants (40.52%) (Figure 3B) was increased. After 24 and 48 h of nematode addition, the nematode mortality of the WT was 82.35 and 99.5% (Figure 3B), respectively, which decreased to 66.45 and 90.80% (Figure 3B) for the *ΔDHXT1* mutants strain, respectively. Meanwhile, the nematode mortality of the *OEDHXT1* transformants strain at 24 and 48 h did not significantly change compared with that of the WT, which was 82.56 and 98.86% (Figure 3B), respectively.

### 2.5. Analysis of RT-qPCR

**Figure 4:**
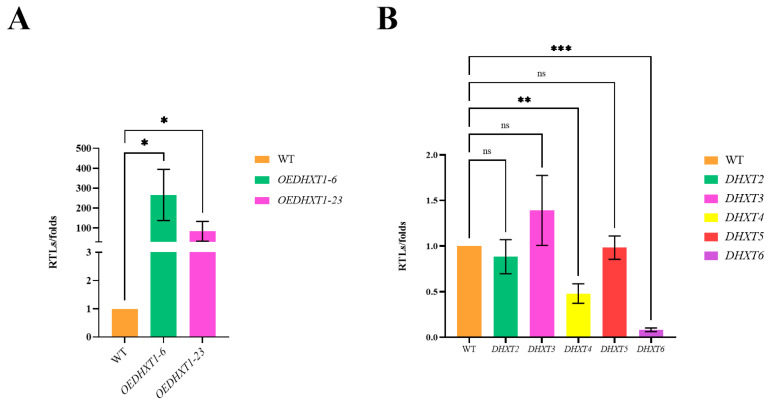
RT-qPCR analysis results. (**A**) Transcript levels of DHXT1 in the *OEDHXT1-6* and *OEDHXT1-23* transformants. (**B**) Transcript levels of other *DHXT2-6* in the *ΔDHXT1* mutants. (ns *p* > 0.05, * *p* ≤ 0.05, ** *p* ≤ 0.01, and *** *p* ≤ 0.001).

The RT-qPCR results showed that *OEDHXT1-6* and *OEDHXT1-23* were overexpressed by 265- and 82-fold (Figure 4A), respectively. In the *ΔDHXT1* mutants, the knockout of *DHXT1* resulted in a decrease in the transcript levels of *DHXT4* and *DHXT6*, although there was no significant difference in the transcript levels of the other three *CAP10*-related genes (Figure 4B).

### 2.6. Metabolomics Analysis of Caenorhabditis elegans Infection by D. haptotyla

**Figure 5:**
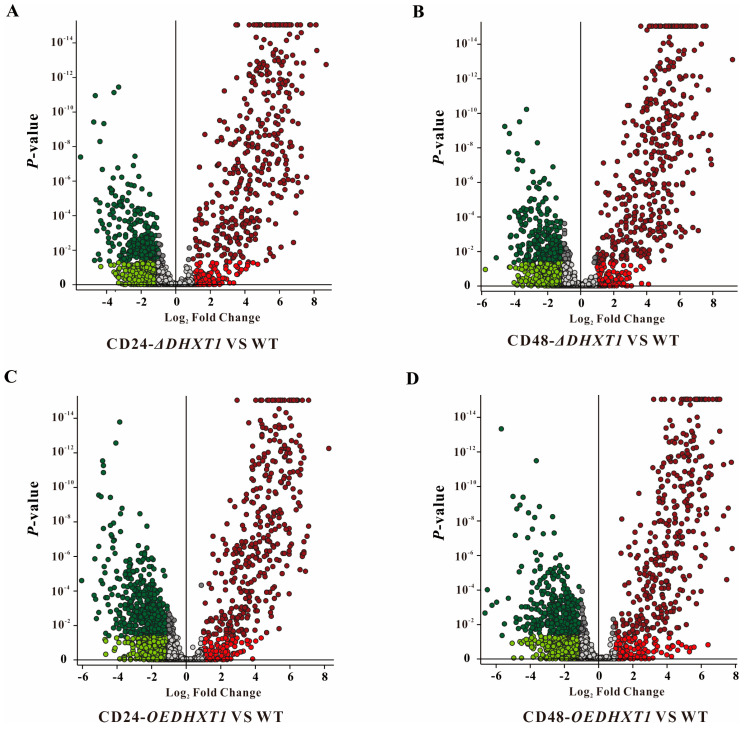
The metabolome analysis during *Caenorhabditis elegans* infection by wild type (WT), *ΔDHXT1* mutants and *OEDHXT1* transformants. (**A**) The volcano plot of CD24-*ΔDHXT1* vs. CD24-WT group. (**B**) The volcano plot of CD48-*ΔDHXT1* vs. CD48-WT group. (**C**) The volcano plot of CD24-*OEDHXT1* vs. CD24-WT group. (**D**) The volcano plot of CD48-*OEDHXT1* vs. CD48-WT group. Significance cutoffs were *p* = 0.05 (Bayes moderated *t*-tests) and fold change (FC) = 1. Each dot represents an individual compound (within ±10 ppm in mass), and the probability of that quantitative observation being statistically significant is indicated by a *p* value on the *y*-axis (determined using the standard linear model within the SIEVE 2.1 software).

To determine whether metabolites and metabolic pathways were altered by *DHXT1* during *Caenorhabditis elegans* infection by wild-type *D. haptotyla*, *ΔDHXT1* mutants and *OEDHXT1* transformants, the extract samples were subjected to LC-MS of untargeted metabolomics. The quantitative analysis of low-molecular-weight metabolites can reveal the relative relationship between changes and metabolites and may indicate metabolite dynamics during the infection of *Caenorhabditis elegans* by *D. haptotyla*. By combining all the analyzed extracts, unique molecular species were detected by UPLC-HR-ESI-MS. The high-resolution MS signals from different isotopes and adduct peaks were combined to ensure that the vast majority of molecular species represented the individual metabolites produced by the corresponding strain. We aimed to determine the differences in secondary metabolites between the CD-*ΔDHXT1* vs. WT and CD-*OEDHXT1* vs. WT groups at 24 and 48 h, respectively. And the data were displayed as a volcano plot, using significance cutoffs of a false discovery rate (FDR)-adjusted *p*-value (<0.05) and a fold-change difference >1. Compared to the wild-type-infected nematode group, in the process of nematode infection by knockout mutants, we observed that 403 metabolites were upregulated, 277 metabolites were downregulated in CD24-*ΔDHXT1* vs. WT (Figure 5A), 414 metabolites were upregulated and 277 were downregulated in CD48-*ΔDHXT1* vs. WT (Figure 5B). Meanwhile, compared to the wild-type-infected nematode group, in the enhancer transformant infection of nematode groups, we observed that 364 metabolites were upregulated, 526 metabolites were downregulated (Figure 5C), 425 metabolites were upregulated and 439 metabolites were downregulated in CD48-*ΔDHXT1* vs. WT (Figure 5D).

## 3. Discussion

The capsular thickness correlates with the expression of *CAP10*, a key gene for viral capsular formation. In *C. neoformans,* the disruption of *CAP10* leads to the loss of pod membranes and the loss of pathogenicity in animal models [28]. As in the CAP10 protein, DHXT1 possesses a structural domain of the glycosyltransferase 90 family and shares similarities with other homologs of filamentous fungi. In this study, the biological functions of *DHXT1* in *D. haptotyla* were explored by the knockout and overexpression of *DHXT1*, phenotypic observations and metabolomic analysis.

The disruption of *DHXT1* resulted in slightly slower growth of mycelia, whereas the overexpression of *DHXT1* slightly accelerated its growth (Figure 1A,B). Furthermore, the overexpression of *DHXT1* resulted in a substantial reduction in aerial mycelia (Figure 1A). These results indicate that *DHXT1* serves a role in the growth and development of mycelia. However, *DHXT1* had no effect on the germination of conidia or on resistance (Figure 2A–D and Figure S2). Similarly, the loss of *CAP10* did not affect the sensitivity of the *C. neoformans* to the oxidant H_2_O_2_ and the cell wall disruptor Congo red [28]. It follows that *DHXT1* does not participate in the regulation of *D. haptotyla* sensitivity to various stress conditions.

NTF specializes mycelia into traps to capture nematodes and obtain nutrients from them [17]. Traps can form spontaneously as well as being induced by signals such as oligotrophic environmental conditions or by certain compounds secreted by nematodes [17]. NTF trap formation may be a highly complex biological process involving numerous genes and pathways [17]. We found that *DHXT1* exerts an important role in trap formation. At 12, 24 and 48 h after nematode addition, the wild type produced 130, 255 and 639 knobs/cm^2^ (Figure 3A), respectively, and the *ΔDHXT1* mutants had 19, 50 and 73 knobs/cm^2^ (Figure 3A), respectively, whereas *OEDHXT1* transformants had 839, 995 and 1761 knobs/cm^2^ (Figure 3A), respectively. The results of the above experiments indicated that the deletion of *DHXT1* resulted in a substantial reduction in traps, while the number of traps increased substantially after the overexpression of *DHXT1*. This suggests that *DHXT1* performs an essential role in the formation of knobs of *D. haptotyla*. It is noteworthy that the nematode mortality of the wild type was 99.5% (Figure 3B) at 48 h after nematode incorporation, whereas the nematode mortality of *ΔDHXT1* (90.8%) (Figure 3B) was not substantially reduced by the reduction in traps. In particular, the *OEDHXTI* transformants exhibited no difference in nematode mortality (82.35% and 99.53%, respectively) (Figure 3B) from that of the wild type (99.53% and 98.86%, respectively) (Figure 3B), although the number of traps increased drastically after 24 and 48 h of nematode addition (Figure 3B). Electron-dense bodies and adhesive proteins are pivotal in the trapping of nematodes by traps [6,29]. Specialized enzymes such as proteases and peptidases produced by NTF are involved in the immobilization of nematodes after capture [11], the degradation of nematode cuticles [12], the penetration and colonization of mycelia and the digestion of nematodes [30]. Previous studies have reported that the deletion of *CAP10* in *C. neoformans* resulted in the upregulation of cell-surface virulence factors. *ΔCAP10* mutants showed a 28-fold increase in acid phosphatase and a 5-fold increase in surface laccase compared with the wild type.

Therefore, we hypothesized that although the deletion of *DHXT1* leads to a decrease in the number of knobs in *D. haptotyla*, it may simultaneously increase the production of virulence factors (such as various enzymes) that contribute to the trapping of nematodes, and thus, there is no substantial decrease in nematicidal activity. Similarly, although the number of traps in the *OEDHXTI* transformants increased substantially, the overexpression of *DHXT1,* meanwhile, may also lead to a decrease in virulence factors, and thus, its nematicidal activity did not differ much more than that of the wild type. The metabolomic results revealed that the loss of *DHXT1* led to an increase in metabolites, and conversely, the overexpression of *DHXT1* led to a decrease in metabolites. Due to the current small amount of research on metabolites of *D. haptotyla* and the limitations of metabolite databases [21,22], a large number of compounds cannot be structurally determined. We speculate that *DHXT1* affects the synthesis and content of some metabolites during the fungal infection of nematodes from the changes in the types and relative contents of compounds. In *ΔDHXT1* mutants, the traps of *D. haptotyla* were significantly reduced, and the pathogenicity was also significantly reduced at 24 h. However, the pathogenicity did not show consistency with the decrease in traps after 48 h but had a clear upward trend. Combined with metabolome analysis, we believe that the fungal pathogenicity increased due to the change in the number and relative contents of metabolites. Subsequent exploration of the biological function of *DHXT2-6* in *D. haptotyla* will be followed up.

## 4. Materials and Methods

### 4.1. Strains and Culture Conditions

*D. haptotyla* YMF1.03409 used in this project was deposited at the State Key Laboratory of Conservation and Utilization of Biological Resources and the State Key Laboratory of Microbial Resources. *D. haptotyla* was routinely maintained on potato dextrose agar (PDA) plates at 28 °C. *Caenorhabditis elegans* N2 strains were maintained at 20 °C on nematode growth media (NGM) plates and fed with concentrated *Escherichia coli* OP50. Tryptone glucose (TG, 10 g of tryptone, 10 g of glucose and 15 g of agar per 1 L of distilled water), tryptone yeast-extract glucose agar (TYGA) (10 g of tryptone, 10 g of glucose, 5 g of yeast extract, 5 g of syrup and 15 g of agar per 1 L of distilled water), TB_3_ (206 g of sucrose, 3 g of yeast extract, 3 g of tryptone and 7.5 g agar per 1 L of distilled water) and WA (1.5% agarose) plates were used in this research.

### 4.2. Bioinformatic and Phylogenetic Analyses of DHXT1

Based on the CXT1 in *Cryptococcus neoformans* (XP_568018.1) and rumi enzymes in *Drosophila* (NP_651095.1), CXT1 homologous proteins were retrieved from the *D. haptotyla* genome using blastn (https://blast.ncbi.nlm.nih.gov/Blast.cgi, on 28 April 2023). The structural domains of the candidate proteins were analyzed by NCBI (https://www.ncbi.nlm.nih.gov/, on 28 April 2023). The phylogenetic relationships of all CAP10 homologs protein were calculated and phylogenetic trees were constructed using MEGA X 10.2.2 software using the maximum likelihood (ML) method with the Bootstrap test setup 1000 times.

### 4.3. The Knockout and Overexpression of DHXT1

Paired primers (Table S1) for the upstream (1820 bp) and downstream (1949 bp) fragments of the *DHXT1* gene (1500 bp) using the genome of *D*. *haptotyla* as a template and the hygromycin resistance gene (*Hyg*) fragment using pCSN44 as a template were designed using Primer5. The amplified upstream, downstream and *Hyg* resistance fragments were ligated to a pRS426 plasmid (enzymatically cleaved by *KpnI* and *BamHI*) using ligase to obtain a *DHXT1* knockout vector (pYUZ603). The knockout fragments using the knockout vector (pYUZ603) as a template were amplified with DHXT1-5 F and DHXT1-3 R primers (Table S1) for protoplast transformation, which was transfected into the receptorized *E. coli* DH5α. The *DHXT1* gene without a terminator and the mCherry fragment were joined with Hi-fusion ligase to a laboratory-held *D. haptotyla gpdA* strong promoter vector (pYUZ87) to obtain an enhancement vector for *DHXT1* (pYUZ601) and transferred into *E. coli* DH5α. pYUZ601 was used as a template with the 601-F and 601-R primers (Table S1) to amplify the overexpression fragment for protoplast transformation.

*D*. *haptotyla* on PDA plates was transferred into TG liquid medium (180 rpm, 28 °C). After two days, the mycelium, filtered out and washed with KTC buffer (1.2 M KCl, 10 mM Tris-HCl, 50 mM CaCl_2_), was treated with snail enzyme (1.6% in KTC buffer, *w*/*v*) and cellulase (1.7% in KTC buffer, *w*/*v*) for 5 h. The enzyme-treated hyphae were filtered, and the filtrate was collected and centrifuged (3000 g, 4 °C) for 6 min; the supernatant was discarded to obtain protoplasts. The protoplasts were washed twice with KTC and resuspended in 150 μL of KTC, and 10 ng of purified knockout or enhancement fragments was added and gently mixed. After incubation on ice for 40 min, 850 μL of PTC solution (50% PEG 6000 in KTC buffer, *w*/*v*) was added and left at 28 °C for 1 h. The putative knockout and enhancement mutants were finally selected using TB_3_ plates containing 200 μg/mL of hygromycin B and verified by PCR amplification.

### 4.4. Effects of DHXT1 on Growth, Mycelial and Trap Morphology and Conidial Germination of D. haptotyla

The WT, *OEDHXT1* transformants and *∆DHXT1* mutants were inoculated on PDA, TYGA and TG plates and incubated at 28 °C to detect the influence of the *DHXT1* gene on mycelial growth. The septa of different strains after staining with 20 μg/mL of calcofluor white (CFW) were observed under fluorescence microscope. To explore whether the *DHXT1* gene affects the germination rate of conidia, the WT and mutant strains were cultured in PDA plates again until conidia were produced, the conidia were washed down with sterile water, and the conidial suspensions were inoculated in the PDA plates, and the germination rate of conidia of different strains was calculated after 24 h. The experiment was performed in three replicates.

### 4.5. Stress Evaluation

TG plates containing different concentrations of cell wall-disturbing agents (SDS, final concentrations of 0.01, 0.02 and 0.03%; Congo red, final concentrations of 0.03, 0.06 and 0.09 mg/mL), osmotic agents (NaCl, final concentrations of 0.1, 0.2 and 0.3 M) and oxidants (H_2_O_2_, final concentrations of 5, 10 and 15 mM) were prepared for determining the stress resistance of the different strains. The experiment was performed in three replicates.

### 4.6. Determination of the Effect of DHXT1 on Trap Formation and Pathogenicity

The wild type, *OEDHXT1* transformants and *∆DHXT1* mutants were incubated on WA (1.5% of agarose) at 28 °C for 4 days, and about 200–300 *Caenorhabditis elegans* were added into plates to interact with the strains. After 12, 24 and 48 h, the number of traps in each plate and the nematode mortality were calculated. The experiment was performed in three replicates.

### 4.7. Analysis of RT-qPCR

Total RNA extracted from *OEDHXT1* transformants and *ΔDHXT1* mutants with the UE Multlsource Total RNA Miniprep Kit (Uelandy, Suzhou, China) was used as a template for the reversal to cDNA using the HiScript III 1 st Strand cDNA Synthesis Kit (+gDNA wiper) (Nanjing Vazyme Biotech Co, Ltd., Nanjing, China). The cDNA samples were used as the template to detect the transcription of *DHXT1* mutants in the *OEDHXT1* transformants and five other *CAP10*-related genes in the *ΔDHXT1* mutants. A LightCycler^®^ 480 SYBR^®^ Green I Master Kit (Roche Diagnostics GmbH, Germany) was used with a 1.0 μL template (200 ng/μL), 0.6 μL of each primer concentration (10 μM) and water to bring the reaction volume to 20 µL for the RT-qPCR assay. The β-Tubulin gene (EVM0002454.1) was used as an internal reference and transcript levels were calculated by the 2^−ΔΔCt^ method [31]. The RT-qPCR assay was performed using LightCycler 480 II (Roche Diagnostics GmbH, Mannheim, Germany) and data were analyzed using LightCycler^®^ 480 SW 1.5.1. Amplification conditions consisted of pre-incubation at 95 °C for 5 min, followed by 45 cycles of 95 °C for 10 s, 52 °C for 10 s and 72 °C for 10 s. The remaining parameters were the default parameters. All primers used for RT-qPCR assays are listed in the table in the online Supplemental Materials (Table S2). The experiment was repeated in three replicates for each strain.

### 4.8. LC-MS Analyses and Metabolomic Data Statistical Analysis

The WT, *ΔDHXT1* mutants and *OEDHXT1* transformants were inoculated in 9 cm WA (1.5% of agarose) plates and incubated for 7 days at 28 °C, and then, 1000–1500 *Caenorhabditis elegans* were added to each plate as an experimental group, in which there were 15 plates (250 mL of WA) per experimental group. The WT, *ΔDHXT1* mutants and *OEDHXT1* transformants that interacted with the *Caenorhabditis elegans* for 24 and 48 h were collected and marked as CD24-WT, CD48-WT, CD24-*ΔDHXT1*, CD48-*ΔDHXT1*, CD24-*OEDHXT1* and CD48-*OEDHXT1.* The above collected samples were separately centrally immersed in 250 mL of ethyl acetate/methanol/glacial acetic acid = 16:3:1 (*v*/*v*/*v*) for three repeated extractions. The experiment was performed in three replicates. The liquid was collected and evaporated in vacuo to obtain the extracts, and methanol was added to make the final concentration of the extracts at 10 mg/mL. The dissolved extract was filtered through a 0.22 μm filter followed by LC-MS analysis. Untargeted metabolomics analysis was carried out by Compound Discoverer 3.0 software.

Untargeted LC-MS metabolomics was performed on a Dionex UltiMate 3000 LC system (Dionex, Sunnyvale, CA, USA) coupled with a Q-Exactive Orbitrap mass spectrometer (Thermo Scientific, San Jose, CA, USA). All samples were separated on a Thermo Scientific Hypersil GoldTM MS C18 (100 mm × 2.1 mm, Thermo Scientific) with a particle size of 1.9 µm at an LC flow rate of 0.3 mL/min and a column temperature of 40 °C. Mobile phase A was 0.1% formic acid in water, and mobile phase B was 0.1% formic acid in methanol. The 30 min gradient for positive ESI mode was set as follows: 0–3 min, 2% solvent B; 3–20 min, 2–99% solvent B; 20–25 min, 99% solvent B; and 25–30 min, 2% solvent B. The injection volume was 5 μL, and each sample was injected in triplicate. The LC-MS instrument was controlled using Thermo Scientific Xcalibur 4.4 software. Metabolomic data statistical analysis was carried out following our previous method [21].

### 4.9. Statistical Analyses

GraphPad Prism version 9.5.1 (GraphPad Software, San Diego, CA, USA) was used to analyze the acquired data and to create graphs. Significant differences were identified by analyzing the comparison between the control and the treated samples using a t-test analysis of variance (APA *p*-value style: ns *p* > 0.05, * *p* ≤ 0.05, ** *p* ≤ 0.01, and *** *p* ≤ 0.001).

## 5. Conclusions

*DHXT1* identified from *D*. *haptotyla* is involved in mycelial growth and aerial mycelium production. Furthermore, *DHXT1* is highly responsible for the formation of traps and the production of metabolites. The disruption of *DHXT1* leads to a decrease in knobs but an increase in metabolites, whereas the overexpression of *DHXT1* leads to an increase in knobs but a decrease in metabolites. Therefore, the spatiotemporal expression of this type of virulence gene can be used as a reference in the subsequent screening of stable and efficient biological control strains, which provides a theoretical basis for the application of *D*. *haptotyla* in the biological control of pathogenic nematodes.

## Data Availability

Data is contained within the article or Supplementary Material.

## Supplementary Materials

The following supporting information can be downloaded at https://www.mdpi.com/article/10.3390/ijms25137384/s1.
