# Supplementary material for: DHXT1, a Virulence Factor of Dactylellina haptotyla, Regulates Pathogenicity by Participating in Trap Formation and Metabolite Synthesis
Source: Int J Mol Sci. 2024 Jul 5;25(13):7384. doi: 10.3390/ijms25137384 (PMC11242603; doi:10.3390/ijms25137384)
Supplement: Supplementary file 1 [file ijms-25-07384-s001.zip › ijms-3058911-supplementary.pdf]

Supplementary Materials for

DHXT1, a Virulence Factor of *Dactylellina haptotyla*, Regulates Pathogenicity by Participating in Trap Formation and Metabolite Synthesis

Xing-Fu Wen †, Ting-Ting Shi †, Ya-Qi Zhang, Si-Han Wang, Chun-Mei Xiang and Pei-Ji Zhao *

State Key Laboratory for Conservation and Utilization of Bio-Resources in Yunnan, School of Life Sciences, Yunnan University, Kunming 650091, China

* Correspondence: pjzhao@ynu.edu.cn

† These authors contributed equally to this work.

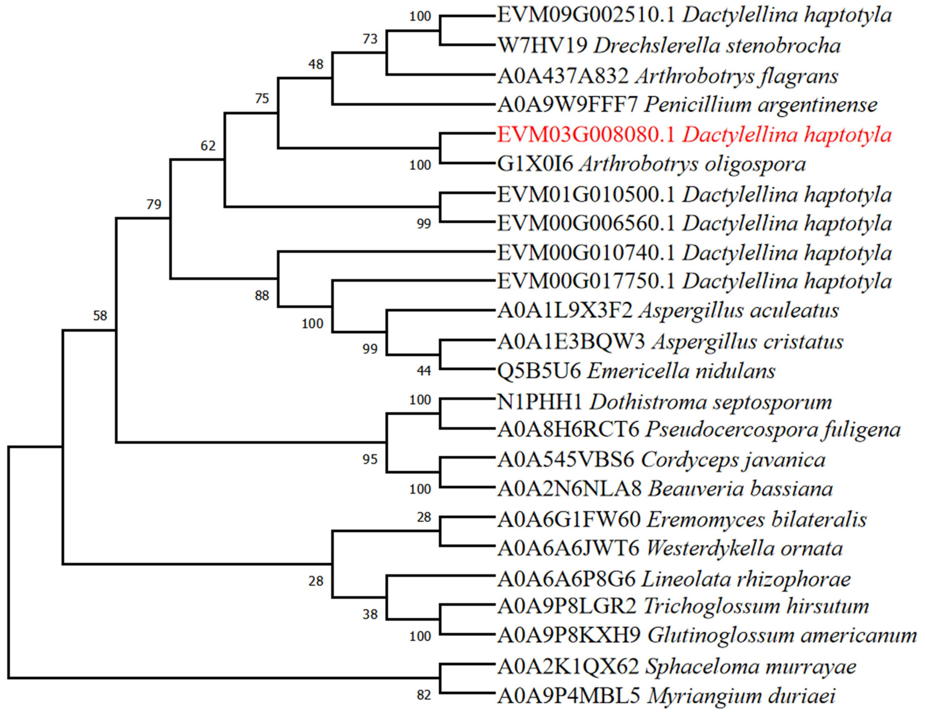

Supplementary Figure S1. A maximum likelihood phylogenetic tree of the sequences orthologous to CAP10 from different fungi**.**

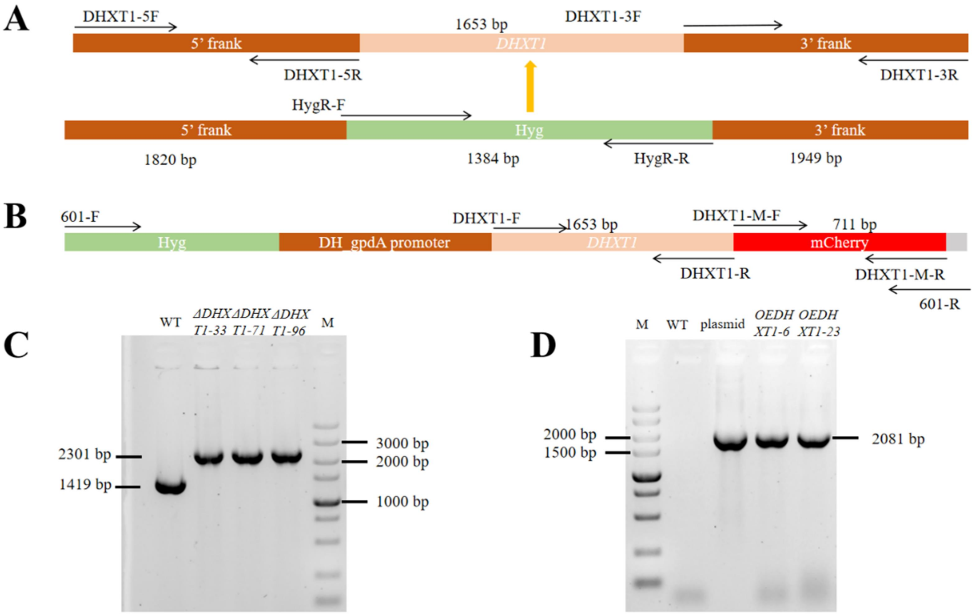

Supplementary Figure S2. Verification of the *ΔDHXT1* mutants and *OEDHXT1* transformants. (A) Diagrammatic sketch of homologous recombination of *DHXT1*. (B) Diagrammatic sketch of overexpression of *DHXT1*. (C) Positive knockout transformants were verified by PCR amplification. M, DNA ladder. WT, the wild-type strain of *D. haptotyla*; *ΔDHXT1-33*, *ΔDHXT1-71* and *ΔDHXT1-96*, the *DHXT1* deletion mutants. (D) Positive overexpression *DHXT1* transformants were verified by PCR amplification. M, DNA ladder. WT, the wild-type strain of *D. haptotyla*; *OEDHXT1-6* and *OEDHXT1-23*, the *DHXT1* overexpression transformants.

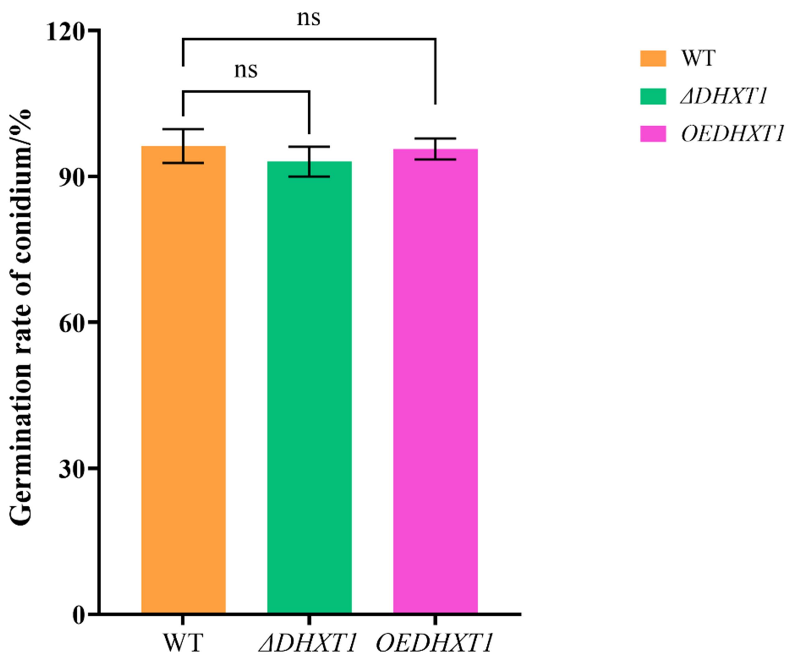

Supplementary Figure S3. The germination rate of conidium of the WT, *ΔDHXT1* mutants and *OEDHXT1* transformants on PDA after 12 h. (ns $p > 0.05$, $*p \leqslant 0.05$, $**p \leqslant 0.01$, and $***p \leqslant 0.001$)

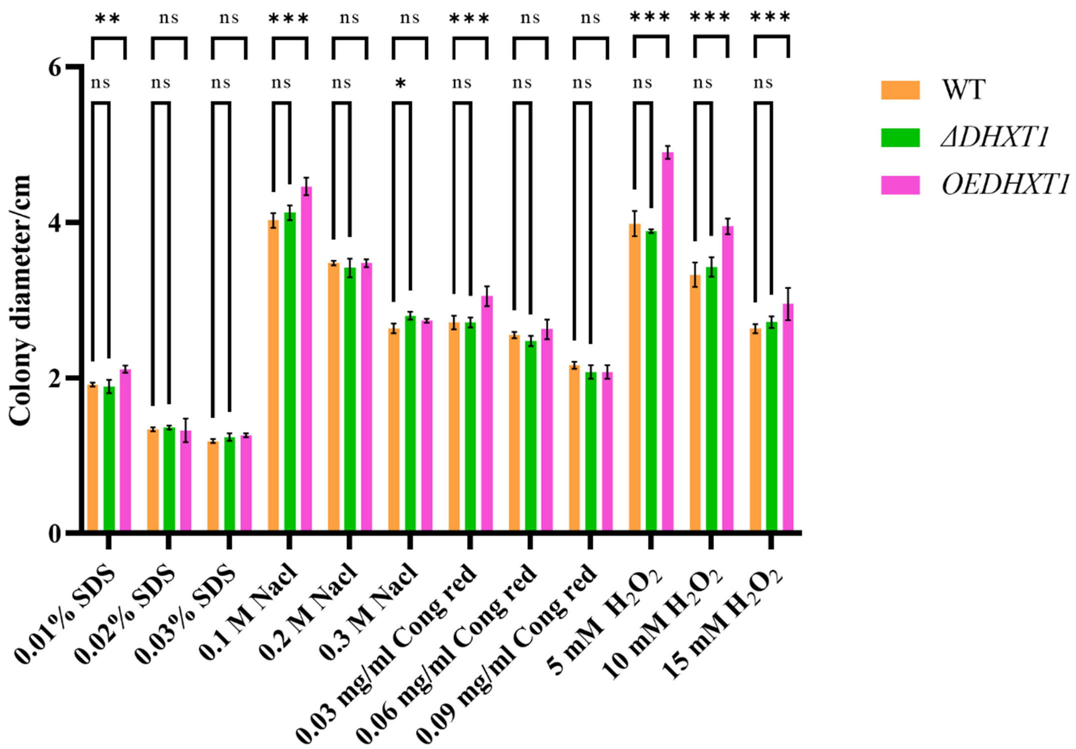

Supplementary Figure S4. Growth of WT, *ΔDHXT1* mutants and *OEDHXT1* transformants on TG plates containing different concentrations of NaCl, H_2O_2, Congo red, and SDS for 8 days. (ns $p > 0.05$, *$p \leqslant 0.05$, **$p \leqslant 0.01$, and ***$p \leqslant 0.001$)

Supplementary Table S1. List of primers used in the knockout and overexpression of *DHXT1*.

Primers	Sequence (5′-3′)	Description
DHXT1-F	ACCGTCAACGATCCTCTAGAATGGTGGCCCGGAGTTTA	Amplify the *DHXT1* gene
DHXT1-R	TCCTCGCCCTTGCTCACCATCGGCAGGTCGTAGTCTAACA	
DHXT1-M-F	TCTGTTAGACTACGACCTGCCGATGGTGAGCAAGGGCGA	Amplify the mCherry fragments
DHXT1-M-R	CTTGCATGCCTGCAGGTCGACCTACTTGTACAGCTCGT	
DHXT1-5F	ATAGGGCGAATTGGGTACCTGGGCTTCTTGGACGGAATCCGAAC	Amplify the *DHXT1* gene 5’ flank
DHXT1-5R	ATCATCTTCTGTCTCCGACAGGGGCTCAAGAGGTTGTTTGAG	
DHXT1-3F	CGTTACTGAAATCTCCAACTCCCCCACCATGGCTCTTTACCAGCA	Amplify the *DHXT1* gene 3’ flank
DHXT1-3R	CGCTCTAGAACTAGTGGATCCAACTTACGGGGCGGGAATAGTAG	
HygR-F	GTCGGAGACAGAAGATGATATTGAAGGAGC	Amplify the *Hyg* resistance fragments
HygR-R	GTTGGAGATTTCAGTAACGTTAAGTGGAT	
603-F	GCGCGTTGTTACAGAACTGCAACATTCG	Verify the knockout mutants
603-R	CAAAGACTGGTATAACTACGCGGTAGCG	
603-F1	ATCGCCAGACAACATGTTTCCTGCGCAA	
603-R1	GAGGATCACGTTGCTCTAACTCAAAGCC	
603-F2	AGTAAGTATGCAAGTGGCTACGGCAGGT	
603-R2	GCCTGGTCCCTGGAAAATCGACACTATA	
601-F	CGGACGTTTTTAATGTACTG	Amplify the overexpression fragments
601-R	TCTTAAAGCTTGCATGCCTG	
DHXT1-oe-F	AATCTTGACGCCCTACCA	Verify the overexpression transformants
DHXT1-oe-R	AGCTTCAGCCTCTGCTTG	

Supplementary Table S2. Paired primers of *CAP10*-related genes used for RT-PCR analysis.

Primers	Sequence (5′-3′)
4583-F	ATCAGGAGTGTGTGAACG
4583-R	GGGTATAACGATTGGTGC
4939-F	TCATGACCGAGAAGCAGT
4939-R	ATGAGGGTGGCAAATCCAC
6975-F	AGGCATTGGATAGCGATG
6975-R	GCTTGGATATCCTCCCAA
7058-F	CAGAAACAGTGTCGGAAG
7058-R	TTTGTGGACTTGGTGGAG
6956-F	GCTCGAAGTCTGTGAGAT
6956-R	ATCCACCGTCTTGTTGTC
DHXT1-RT-F	GGAGCTCCTGTTATAGACGA
DHXT1-RT-R	CTATGCACGAACGGTCTCAT
β-tubulin-F	GATGGCTCCGGTGTCTA
β-tubulin-R	CAGTTGTTACCGGCGC
